# A pilot study of semiquantitative treatment evaluation following nonthermal atmospheric‐pressure plasma administration for onychomycosis

**DOI:** 10.1111/srt.13263

**Published:** 2022-12-29

**Authors:** Hye‐Jin Ahn, Tae‐Eun Kim, Ye‐Jin Lee, Su Jin Jeong, Min Kyung Shin, Ki‐Heon Jeong

**Affiliations:** ^1^ Department of Dermatology Kyung Hee University Medical Center Seoul South Korea; ^2^ Medical Science Research Institute Kyung Hee University Medical Center Seoul South Korea

Various methods have been used to treat onychomycosis, including application of topical and systemic antifungal agents and laser therapy. In current standard systemic treatment, the complete cure rate of toe nails is 38% with terbinafine and 14% with itraconazole.^1^ In addition, recurrences (i.e., relapse [same infection after incomplete cure] or reinfection [same infection after complete cure]) occur at a rate of 20–25%.^1^ Near‐infrared diode lasers, 1064 nm neodymium‐doped yttrium aluminum garnet (Nd:YAG) lasers, dual diode lasers, 1320 nm Nd:YAG lasers, and fractional CO_2_ lasers have all been used for the treatment of onychomycosis.^2^ A recent review reported that 1064 nm Nd:YAG laser treatment resulted in lower mycologic cure rates (11%) than oral and topical therapies approved by the Food and Drug Administration (29–61%).^2^ Thus, an effective and safe local target therapy for onychomycosis is needed. Plasma lasers have antibacterial and antifungal effects; these lasers are generated in the air by a strong electric field pulse generated by the ionization of air molecules into ozone, hydroxyl radicals, and nitric oxide, which exert antifungal properties.^3^ In previous studies, nonthermal atmospheric‐pressure plasma (NTAP) inhibited the growth of *Trichophyton rubrum* in vitro,^4^ achieving an overall clinical cure rate for infected toenails of 53.8%, and a mycological cure rate of 15.4% in an in vivo pilot study.^5^

This study aimed to evaluate the feasibility of NTAP as a treatment option for onychomycosis. A total of five participants (four females and one male, mean age 46.4) with toenail onychomycosis provided informed consent to participate. Reflectance confocal microscopy (RCM, VivaScope^®^ 1500; Lucid Inc., Rochester, NY, USA) was performed on the infected toenails before and immediately after NTAP (PLADUO; SHENB, Seoul, Korea), which generated plasma from argon and nitrogen gas sources, delivering pulses to the skin. The NTAP was applied to the affected nail plate with pulse energy 1.5 J, frequency 3 Hz of nitrogen gas and followed by 0.75 J, 3 Hz of argon gas, three passes, respectively.

The number of fungal spores and hyphae was counted in each 500 × 500 µm^2^ RCM image. To evaluate fungal spore dispersion, the RCM images were divided in a grid pattern. Wilcoxon signed‐rank test was then applied to compare the total number and dispersion effect of spores before and after NTAP treatment. Statistical significance was set at *p* < 0.05.

Before NTAP treatment, a spherical spore surrounded by a bright visible wall was observed in the RCM image; after NTAP treatment, cell wall disruption and fungal hyphae breakage were observed (Figure 1). The size of fungal spores is reported to range from 2 to 50 µm in diameter.^6^ We measured the size of the fungal spores to be 2−8 µm using Image J. The spores were clustered; however, immediately after treatment, the spores became more disperse (*p* = 0.5), and the total number of spores decreased from 216 to 138 (*p* = 0.31) (Figure 2), although this difference was not statistically significant. On the RCM image, crack‐like changes in the nail plate were seen after treatment, which was thought to indicate a synergistic effect when combined with other treatments such as topical antifungal agent (Figure 3).

**FIGURE 1:**
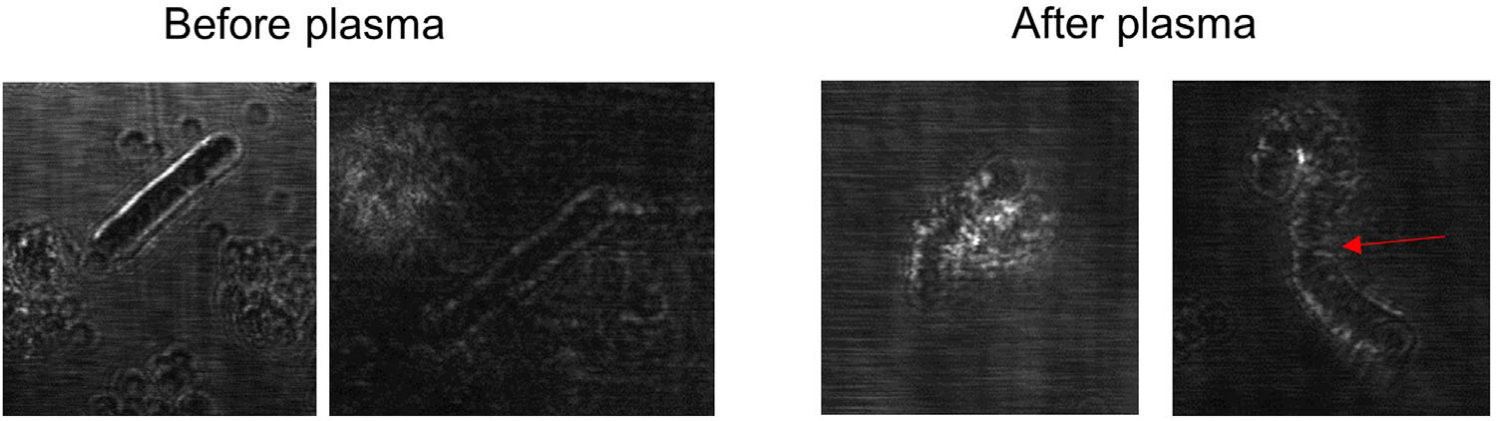
A hyphae with an intact cell wall was observed on reflectance confocal microscopy (RCM) image. After plasma treatment, disruption of the cell wall and broken hyphae (red arrow) were observed

**FIGURE 2:**
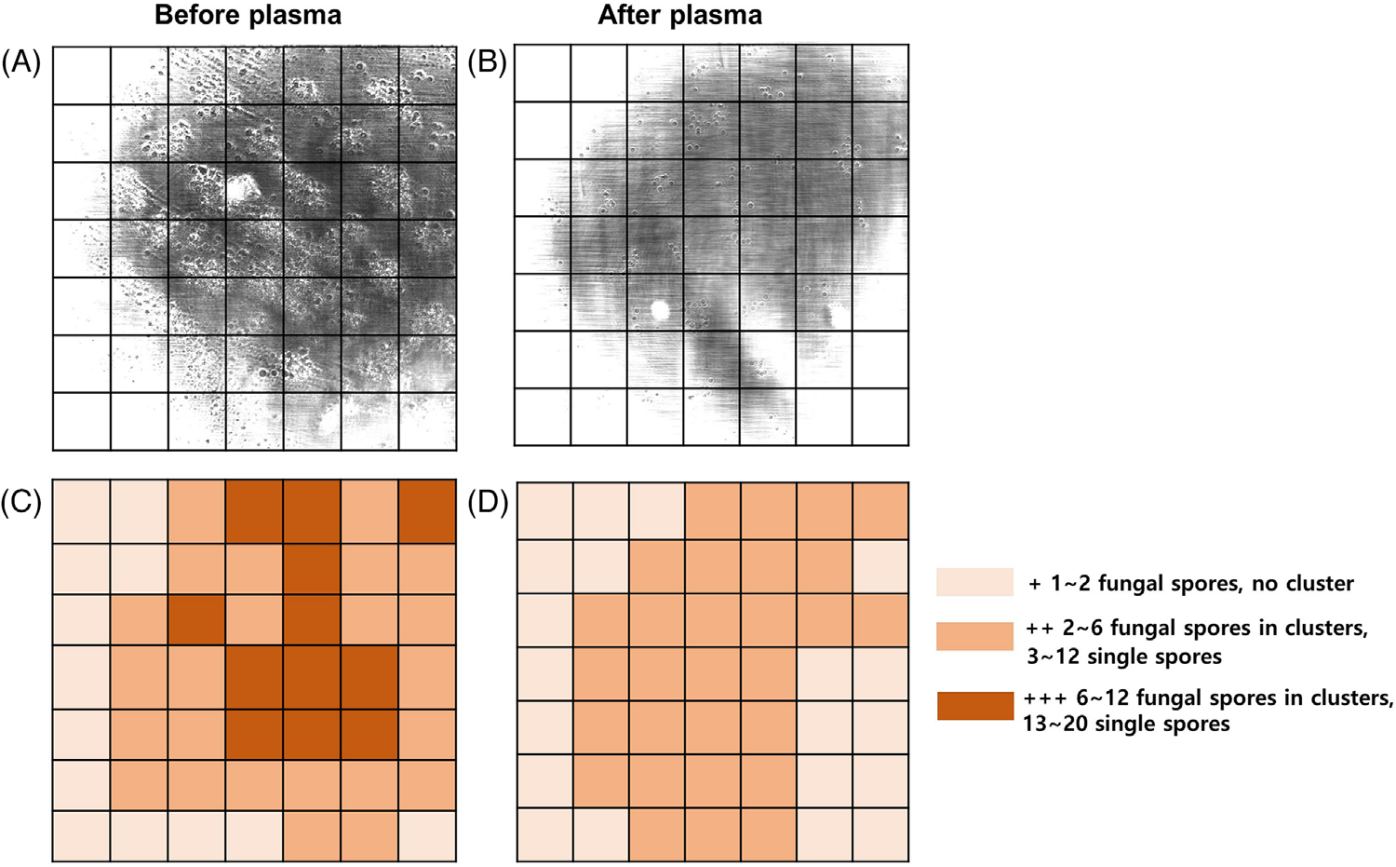
An RCM image (500 × 500 µm^2^) with grid pattern showing that clusters of fungal spores (A) were dispersed (B) following plasma treatment. The mean number of fungal spores of all participants were counted, and the grid pattern showed a decreased density after plasma treatment (C and D)

**FIGURE 3:**
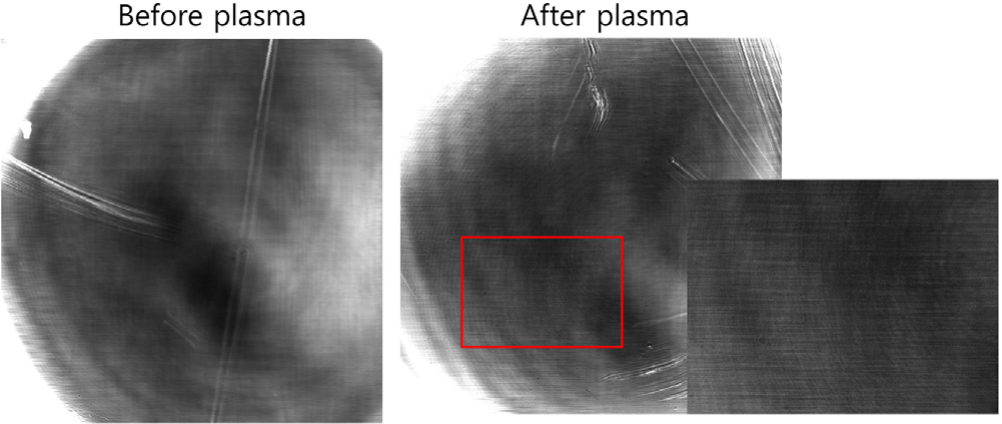
On the RCM image, reflectance was increased and fine lines were clearly visible, reflecting the nail plate changes immediately after treatment

Biofilms are structured sessile microbial communities comprising microorganisms adhering to a surface and each other via an extracellular polymeric matrix.^7^ Similar to bacteria, fungi are also able to form biofilms; *T. rubrum* and *T. mentagrophyte* biofilms have been previously reported.^8^ The adhesion of fungal spores to surfaces occurs readily and is a prerequisite for biofilm formation.^9^ Recent studies have reported the positive effect of plasma on fungal biofilm inactivation.^10^, ^11^ Our prior in vitro study showed destruction of colonies of *T. rubrum* with waxy surface after NTAP irradiation and scanning electron microscopy, confirming that the exopolymeric matrix of the biofilm^8^ disappeared (Figure S1). Despite the small sample size, this study revealed that NTAP treatment disrupted the cell wall and decreased the number and dispersed clusters of spores. These results suggest that NTAP treatment could exert preventive effects against fungal biofilms.

There were no serious side effects during NTAP irradiation, except for a minor heating sensation. To reduce this unpleasant sensation, it is necessary to keep moving the hand‐piece, instead of fixing it at one point. Although the effect may be weak as a single treatment, we suggest that irradiating onychomycosis with NTAP prior to topical antifungal or laser treatment could enhance the therapeutic effect.

## CONFLICT OF INTEREST

The authors declare that they have no conflicts of interest.

## ETHICS STATEMENT

Informed consents were obtained from all participants.

## Supporting information

Supporting Information

## Data Availability

The data that support the findings of this study are available from the corresponding author upon reasonable request.
