# Supplementary material for: A pilot study of semiquantitative treatment evaluation following nonthermal atmospheric‐pressure plasma administration for onychomycosis
Source: Skin Res Technol. 2022 Dec 29;29(1):e13263. doi: 10.1111/srt.13263 (PMC9838757; doi:10.1111/srt.13263)

**Supplementary figure.** In prior *in-vitro* experiment, *T. rubrum* colonies in sabouraud dextrose agar (SDA) plate was destroyed after NTAP (0.576 kV, 69 mA, 81 kHz, Argon gas) irradiation (a). Arrows denote exopolymeric matrix covering the hyphae before irradiation in SEM image. Surface of hyphae became smooth after irradiation (b).

**(a)**


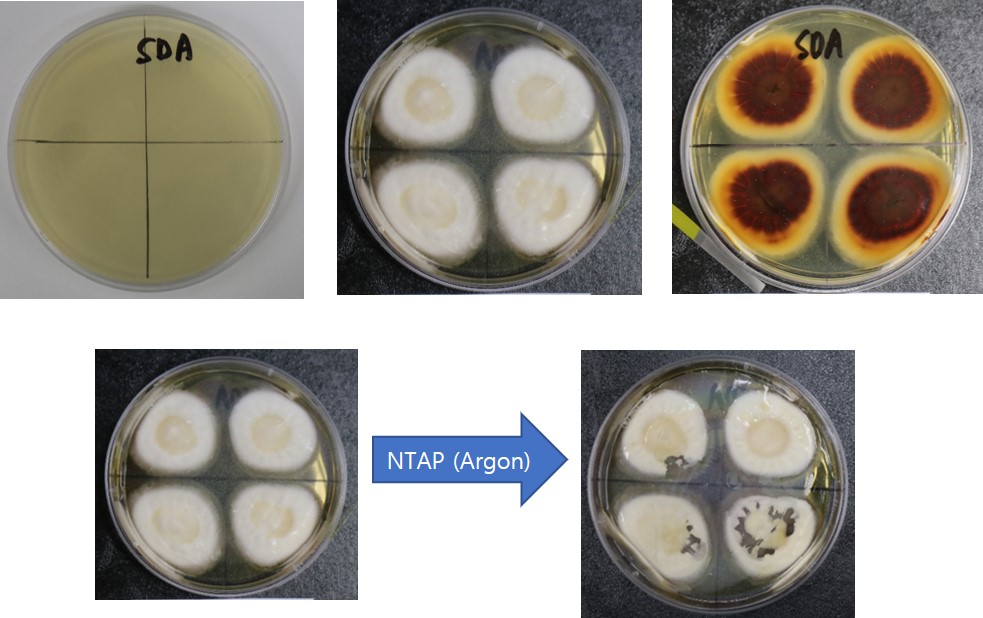


**(b)**


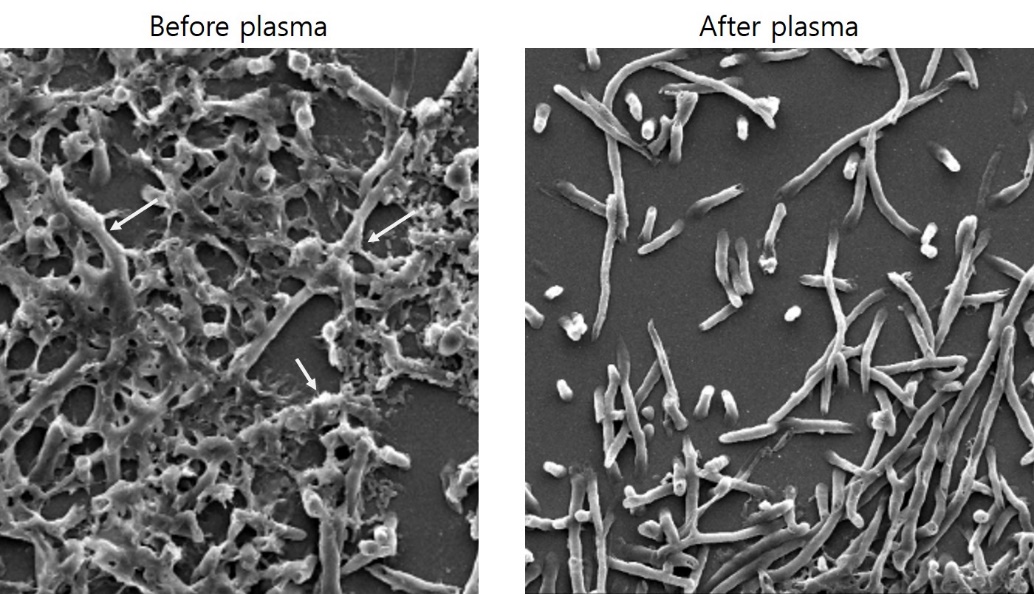

Supplement: Supplementary file 1 — Supporting Information [file SRT-29-e13263-s001.docx]
